# Genome scanning of behavioral selection in a canine olfactory detection breeding cohort

**DOI:** 10.1038/s41598-022-18698-4

**Published:** 2022-09-02

**Authors:** Alexander W. Eyre, Isain Zapata, Elizabeth Hare, Katharine M. N. Lee, Claire Bellis, Jennifer L. Essler, Cynthia M. Otto, James A. Serpell, Carlos E. Alvarez

**Affiliations:** 1 Center for Clinical and Translational Research, The Abigail Wexner Research Institute at Nationwide Children’s Hospital, Columbus, OH 43205 USA; 2 Department of Biomedical Sciences, Rocky Vista University College of Osteopathic Medicine, Parker, CO 80134 USA; 3 Dog Genetics LLC, Astoria, NY 11102 USA; 4 Division of Public Health Sciences, Department of Surgery, Washington University in St. Louis School of Medicine, St. Louis, USA; 5 Human Genomics, Genome Institute of Singapore, Agency for Science, Technology and Research, Singapore, 138672 Singapore; 6 Centre for Genomics and Personalised Health, Genomics Research Centre, School of Biomedical Sciences, Queensland University of Technology (QUT), Kelvin Grove, QLD Australia; 7 Penn Vet Working Dog Center, School of Veterinary Medicine, University of Pennsylvania, Philadelphia, PA 19146 USA; 8 Penn Vet Working Dog Center, Department of Clinical Sciences and Advanced Medicine, School of Veterinary Medicine, University of Pennsylvania, Philadelphia, PA 19146 USA; 9 Department of Clinical Sciences and Advanced Medicine, School of Veterinary Medicine, University of Pennsylvania, Philadelphia, PA 19104 USA; 10 Department of Pediatrics, The Ohio State University College of Medicine, Columbus, OH 43210 USA; 11 Present Address: Department of Anthropology, Tulane University, New Orleans, USA; 12 Present Address: Department of Animal Science, State University of New York College of Agriculture and Technology at Cobleskill, New York, USA

**Keywords:** Genetics, Neuroscience

## Abstract

Research on working dogs is growing rapidly due to increasing global demand. Here we report genome scanning of the risk of puppies being eliminated for behavioral reasons prior to entering the training phase of the US Transportation Security Administration’s (TSA) canine olfactory detection breeding and training program through 2013. Elimination of dogs for behavioral rather than medical reasons was based on evaluations at three, six, nine and twelve months after birth. Throughout that period, the fostered dogs underwent standardized behavioral tests at TSA facilities, and, for a subset of tests, dogs were tested in four different environments. Using methods developed for family studies, we performed a case-control genome wide association study (GWAS) of elimination due to behavioral observation and testing results in a cohort of 528 Labrador Retrievers (2002–2013). We accounted for relatedness by including the pedigree as a covariate and maximized power by including individuals with phenotype, but not genotype, data (approximately half of this cohort). We determined genome wide significance based on Bonferroni adjustment of two quasi-likelihood score tests optimized for either small or nearly-fully penetrant effect sizes. Six loci were significant and five suggestive, with approximately equal numbers of loci for the two tests and frequencies of loci with single versus multiple mapped markers. Several loci implicate a single gene, including *CHD2*, *NRG3* and *PDE1A* which have strong relevance to behavior in humans and other species. We briefly discuss how expanded studies of canine breeding programs could advance understanding of learning and performance in the mammalian life course. Although human interactions and other environmental conditions will remain critical, our findings suggest genomic breeding selection could help improve working dog populations.

## Introduction

In addition to the long term and large body of research on dog behavior^1^, there is a rapidly growing literature focused on behavior and cognition in working dogs^2^. This is in part due to interest in the evolutionary biology of selected traits such as herding, but also to increasing needs for various types of assistance functions, such as guide dogs for blind people; and for police, military, and security working dogs for purposes including olfactory detection and deterrence. The average cost of a trained service dog is $15,000–$30,000 (Ref. ^3^), and working dogs at US federal agencies can cost approximately $50,000 or more. As demand for such dogs grows, there is increasing interest in improving their training success rate and performance^4^. Historically, most research on working dog training has studied behavior and temperament, but cognitive analyses are becoming more common and functional magnetic resonance imaging in awake dogs has been applied to this question^5^ (reviewed by^2^).

Neuroscience has recently experienced an explosion of progress in several areas, including genetics and brain imaging. However, there are widespread concerns that too much research lacks a behavioral context or is dissociated from biology and psychiatry^6,7^. The emergence of neuroscience apart from neurology in the early 1900s was largely based on experimentation on a great array of diverse species^8^. However, since the 1970s there has been increasing reliance on mouse models. Mice were used in almost half of studies funded by the US National Institutes of Health in 2015, whereas the next three species combined (fruit fly, zebrafish, and the worm *C. elegans*) were funded at a ten-fold lower level. Unlike mice and most other animal models, dogs present many human-like aspects such as membership in human families, living to advanced age, epidemiology, advanced health care and even different types of long-term work experiences^9^. Moreover, the evolutionary history of dogs makes them a uniquely powerful model for the study of complex genetics^10^. Canine model advantages over human investigation include reduced heterogeneity, larger effect sizes of variations resulting from strong positive selection and relaxed negative selection, lack of socioeconomic confounding, and a shorter generation time. Dogs have hundreds of isolated populations or breeds with diverse personality and behavior traits. Recent genome scans in dogs have mapped personality, normal and problem behaviors, and cognitive traits both in single breeds and across diverse breeds^11–15^. In parallel, brain imaging studies have revealed structure–function correlations that suggest neurodevelopmental and physiological potential^16,17^.

For the reasons mentioned above, dogs are an ideal model for identifying genetic variation broadly associated with learning, defined as a change in an individual’s behavior or abilities resulting from experience; and work performance, defined for our purposes here as an individual’s effectiveness at doing a job well. It is widely agreed that working dog success reflects the maximal matching of breed physical and behavioral conformation with the necessary performance criteria (incl. the lowest rate of excluding faults). The basis for this is that dogs work because they find the activity inherently rewarding^18^. Retrievers have the desired size and agility, and variation of the predatory motor pattern sequence: accentuating searching orientation; directly going to accentuated grabbing-biting without first proceeding through eyeing, stalking, and chasing; and never continuing to kill-biting. However, selection of optimal breed conformations for different work is not sufficient. Success also requires proper development during the early developmental period and both general and specific environmental exposures. All working dogs need socialization with humans, sled dogs require socialization with other sled dogs, and hunting dogs require exposure to guns firing in their first year of life. Genetic and environmental factors can influence these types of success in humans and other animals. Those influences can involve behavior, temperament, cognition, and their interactions. For instance, the prevalence of attention deficit hyperactivity disorder (ADHD) in humans is ~ 4% and its most common comorbidities are learning disabilities (45%), anxiety (38%) and other behavioral (31%) disorders^19^. Natural hyperactivity, impulsivity, and inattention as in human ADHD are common in dogs, vary in frequency and severity across breeds, and are highly comorbid with fearfulness, aggressiveness, and compulsive behaviors^20^. Longitudinal data from working dog breeding programs present an opportunity to advance knowledge of effects that influence success in early behavioral development, preselection for training (*the subject of this work*), training, and work performance and longevity.

The following three examples show recent progress in predicting success of working dog training. The first study measured cognitive skills and temperament from birth to adulthood in Labrador and Golden Retrievers, and German Shepherd Dogs from The Seeing Eye breeding and training program^21^. The traits most predictive of successful training were faster solving of a multistep task and lower levels of a type of anxiety (*lower* maternal behavior predicted success but is not consistent across all working dogs). A second study used a battery of 25 cognitive tests on independent samples of retrievers trained for assistance or olfactory detection^22^. The main finding was that the different work types of the two populations were reflected in different cognitive tests being the most predictive of success. The third study used retrievers trained for assistance work^23^. It determined predictive performance by modeling behavioral assessment scores from instruments designed to identify problematic behaviors in pet and assistance dogs. Unsurprisingly, that third study assessing problem behaviors was far better at predicting dogs that failed whereas the first two studies focused on cognition were far better at predicting success.

The present work addresses what may be referred to as preselection of dogs for subsequent training as working dogs. Generally, such preselection takes place in the context of requests for and in-person brief evaluation of dogs for purchase based on breed, medical, and behavioral criteria. However, this work is a study of a Transportation Security Administration (TSA) detector-dog breeding and training program in which dogs were fostered for 15 months and observed and tested for performance-related behavior at 3, 6, 9 and 12 months (in a period ending in 2013). Several of those tests are dependent on odor detection, including finding objects by olfaction. Others measure other traits such as interest in possessing a toy or playing tug of war. Specifically, we used a multigenerational TSA cohort of 528 Labrador Retrievers to perform a genome scan of behavioral risk of elimination prior to entering training. Studies of the validity of the behavioral testing in this TSA program^24,25^ and, by the same investigators, in the Australian Border Force Detector dog program^26^ that overlapped our time-period have been published; but this is the first genetic study. For the few loci in which only one gene is primarily implicated, the biological relevance is consistent with behavior. We discuss the potential of such studies to improve the working dog population^4^ and advance the understanding of complex behavioral systems.

## Results

### Cohort and population structure analysis

We used a multigenerational population from the TSA detector dog breeding and training program to map elimination due to behavioral reasons before commencing training (Table 1). In that pretraining period, dogs were tested for behavior at three, six, nine and twelve months, both at TSA facilities and in four different environments^25^. We accessed behavioral testing data and biosamples collected from four generations of related Labrador Retrievers from 2002–2013. That data also included whether puppies were selected for training and, if not, whether they were eliminated for medical or behavioral reasons. All breeding Labrador Retrievers were sourced from US breeders of dogs with hunting titles (US) or the Australian Customs Detector Dog Program (Aus. Customs Service, ACS). Breeding Labrador Retrievers were otherwise undocumented to us except that they did not include dogs with brown coat color (associated with show vs. working dog status^27^) and that ACS dogs presumably were part of a breeding and selection program initiated in 1993^28^. The ACS detector dog program followed a breeding and selection program for guide dogs that was initiated by the Royal Guide Dogs Associations of Australia (RGDAA) in 1964 and shortly after was joined by the University of Melbourne. The RGDAA program and Kadnook Kennels (Aus.), a key source of their dogs, provided the base population of the detector dog program. That guide dog population also contained contributions from UK and US dogs referred to as “outside” stock (extent unknown)^28^. At least in its early phase, the detector program received dogs determined to be unsuited for the guiding program. The selection goals for the detector program were referred to as “to provide a steady supply of dogs suitable for training; dogs with a stable temperament, free from genetic disorders and with a long and healthy working life”^28^. The founders and dogs produced in our TSA cohort collected through 2013 were 74.3% US, 5.6% ACS, 18.0% US x ACS, and 2.1% of unknown source. The pedigree of the full cohort shows a greater risk of elimination for behavioral reasons among dogs most closely related to the founder population (Suppl. Fig. S1). The TSA pretraining program for each dog spanned approximately 12 months after which the dogs were either accepted into training (58.9%) or eliminated for medical (17.6%) or behavioral (23.9%) reasons. Behavioral testing data were available for 528 dogs, of which 296 had biosamples used for genome wide SNP genotyping (~ 173 k SNPs, Illumina CanineHD). After quality filtering, the final genome wide SNP set contained ~ 112 k markers.

**Table 1:** Case/control information for dogs in the 2013 TSA cohort.

	Genotyped Dogs	No Genotype
Successful	267	180
Eliminated	29	97
Unknown	0	45
Total Dogs	296	322
Male	158 (17)	147 (44)
Female	138 (12)	130 (53)

**Figure 1:**
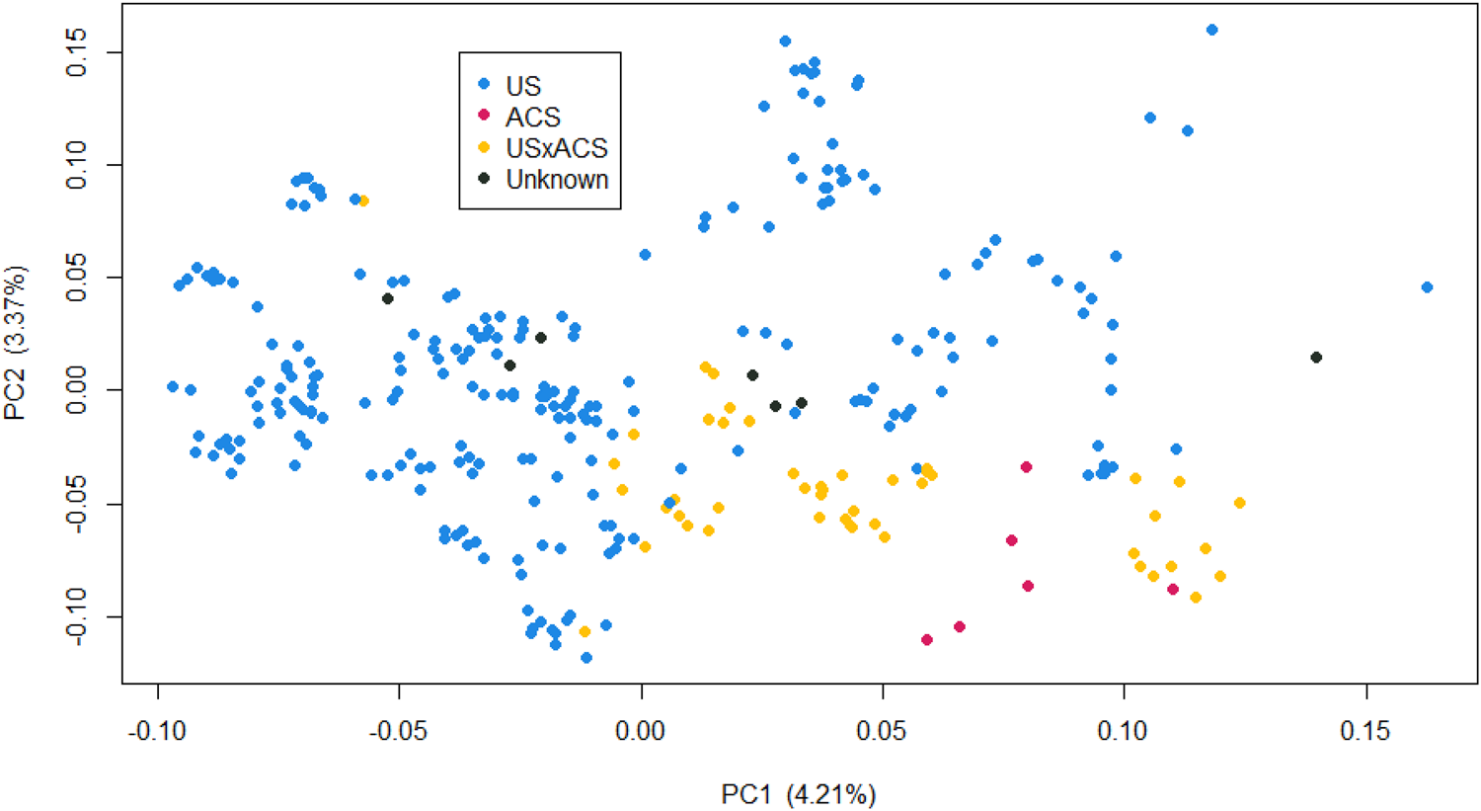
Principle Component Analysis of genotyped Labrador Retrievers. Principal component 1 (PC1) plotted against PC2. The value in the parentheses represents the % of variance explained by the component.

We performed principal component analysis (PCA) of the genotyped Labrador Retrievers to identify population structure (Fig. 1). Both PC1 and PC2 showed a slight separation between the US and ACS Labs, and the expected intermediate location of the US x ACS crosses. Visualizing additional PCs did not reveal any further separation between the groups. STRUCTURE model-based clustering analysis^29^ of the same genotypes failed to detect more than one population when run using both admixture and linkage models over a range of K values and burnings/MCMC reps.

### Genome scan for risk of elimination due to behavior

**Figure 2:**
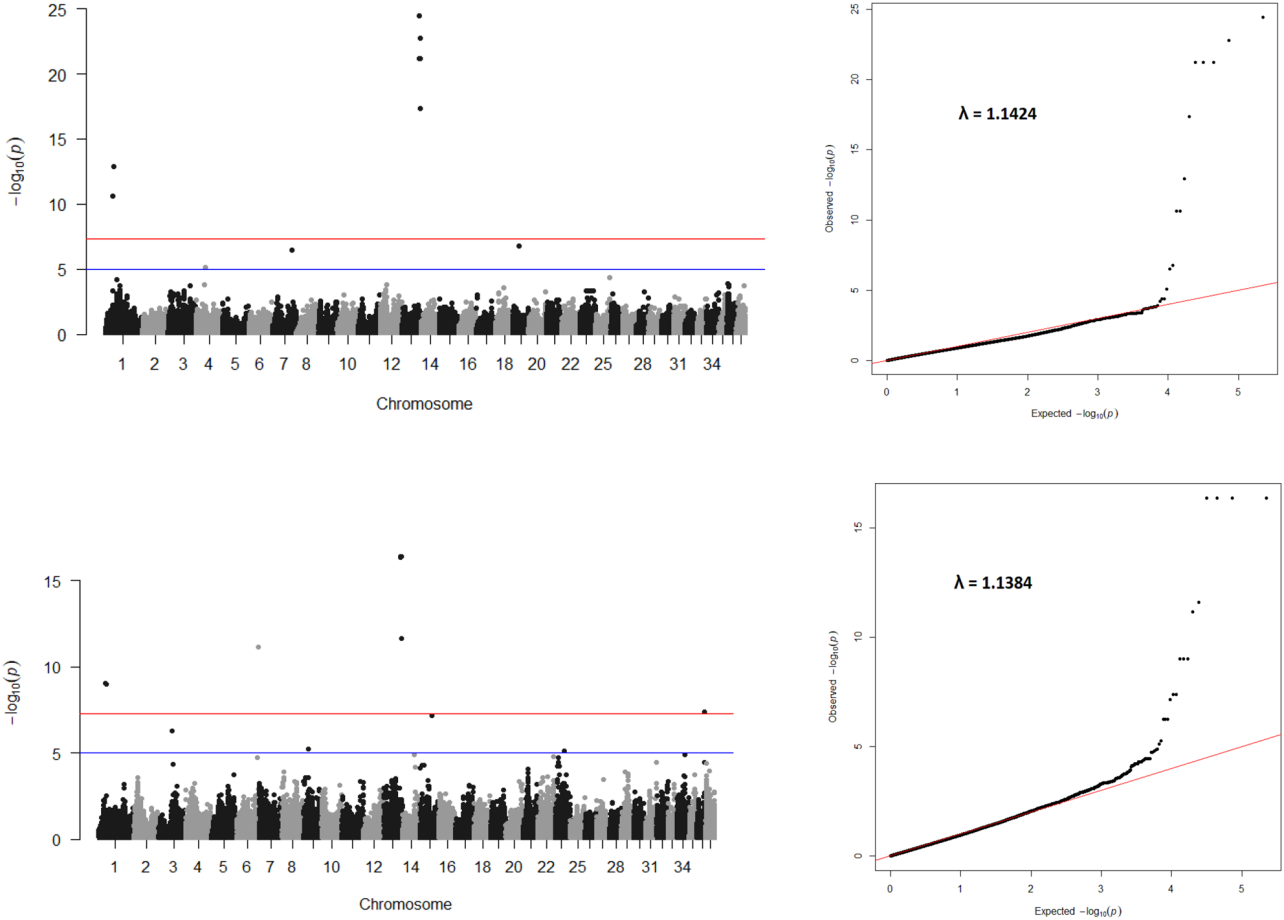
Manhattan and QQ Plots of ROADTRIPS2 output. A Bonferroni cutoff of 2.2 × 10^−7^ for significant hits and 5 × 10^−5^ for suggestive hits is mapped. (**A**) RM output. (**B**) RW output.

We used ROADTRIPS2 for genome wide association because it controls for both population structure and relatedness, and increases power by including dogs with phenotype but no genotype^30^. It makes use of kinship information calculated from the pedigree and an imputation procedure that accounts for the relatedness of individuals. The program calculates three statistics. As the original ROADTRIPS work did, we designed our study to use two of those and to correct for multiple testing for all tests. RM is an extension of the M_QLS_ test that uses pedigree-based weights to improve power and is optimal for two-allele disease models with small effect sizes. RW is an extension of the W_QLS_ test, which accounts for correlation among related individuals by incorporating optimal weights based on pedigree information and is optimal for rare allele disease models that are close to fully penetrant. Genome wide significance was based on Bonferroni adjustment (P ≤ 2.2 × 10^−7^) and suggestive significance was arbitrarily set at P ≤ 1 × 10^−5^. We performed GWA for elimination due to behavioral reasons and identified six significant and five suggestive loci (Fig. 2; Table 2). The λ inflation factors for the two tests were slightly above the 1.11 generally considered benign. The Q-Q plots showed population structure and relatedness were well controlled. That is consistent with the robust control of type 1 error due to population structure and family relatedness demonstrated in studies of ROADTRIPS^30^.

**Table 2:** Genome scan for behavioral elimination in 2013 TSA cohort.

SNP (CanFam3.1)	GWA RM^1^	GWA RW^1^	Risk Allele	f (Acc.)	f (Elim.)	Genes^2^	Brain relevance of top positional candidates (GWAS Catalog and cited sources)
chr1.22989459	**2.39E−11**	**9.46E−10**	G	0.985	1.000	12 genes (incl. SMAD4, MC2R, MC5R; peak near CCN2/CTGF)^3^	NA
chr1.24927539	**2.39E−11**	**9.46E−10**	G	0.985	1.000		
chr1.25289424	**1.23E−13**	**9.57E−10**	A	0.983	1.000		
chr3.47103534	8.18E−01	*5.47E−07*	G	0.927	0.966	**CHD2**	Human GWA brain traits include several cognitive, schizophrenia, self-injurious behavior. There is extensive knowledge of CHD2 in brain biology and pathophysiology in humans and mice
chr3.47134935	8.54E−01	*5.47E−07*	G	0.929	0.966		
chr3.47212502	8.18E−01	*5.47E−07*	G	0.927	0.966		
chr4.31420247	*7.96E−06*	9.08E−03	G	0.948	0.966	**NRG3**	Human GWA traits incl. schizophrenia and drug use. There is extensive knowledge of NRG3 in brain biology and pathophysiology in humans and mice
chr6.76632282	3.66E−03	**6.70E−12**	G	0.951	1.000	DEPDC1-AS1, DEPDC1, LRRC7	LRRC7: human GWA incl. many cognitive traits, attention deficit hyperactivity disorder, drug use
chr7.66358701	**3.25E−07**	6.64E−02	G	0.948	0.919	**ABHD3**^Note #4^	In mouse, highly enriched in brain; highest brain expression in cerebellum in humans and mice (Human ABHD3 introns contain ROCK1 cortex eQTL, ESCO1 thyroid eQTL (GTEx); Mouse ROCK1 dosage affects dendritic spine structure; Human ESCO1 GWA subcortical volume)^4^
chr9.16559175	2.51E−01	*5.60E−06*	G	0.867	0.983	**KCNJ2** (closely followed by KCNJ16)^**5**^	Human KCNJ2 mutations cause Andersen-Tawil syndrome (OMIM170390), which includes a distinct neurocognitive phenotype with deficits in executive function and abstract reasoning, and may also present mood disorders and seizures^5^
chr13.55534649	**6.36E−22**	**4.21E−17**	A	0.976	0.983	54 genes (incl. EPHA5, GNRHR; peak near CENPC1)^6^	NA
chr13.57789399	**3.66E−25**	**4.12E−17**	C	0.974	0.983		
chr13.58498102	**6.29E−22**	**4.18E−17**	G	0.976	0.983		
chr13.59658137	**6.29E−22**	**4.18E−17**	A	0.976	0.983		
chr13.59763520	**4.27E−18**	**2.39E−12**	G	0.976	0.983		
chr13.59902870	**1.64E−23**	2.43E−01	A	0.976	1.000		
chr15.40757218	3.86E−03	**6.86E−08**	A	0.948	1.000	DRAM1^Note #7^	Many human GWA traits of brain volume and structure, incl. white matter microstructure
chr19.21040815	**1.70E−07**	2.56E−02	A	0.938	0.966	ENSCAFG00000004229^Note #8^, PLEKHB2, HS6ST1	HS6ST1 was mapped in human GWA of general cognitive ability and has extensive research literature in multiple areas of neuroscience, including neurodevelopment, effects of stress on gene expression and behavior, and reproductive behavior
chr23.33369350	1.59E−01	*7.71E−06*	A	0.839	0.983	**NCK1**^Note #9^	There is one human GWA brain trait: neuroticism. In mice, loss of NCK1 affects dendritic spine density in the amygdala, associated with abnormal stress response and increased anxiety^9^
chr36.25252101	7.98E−03	**4.05E−08**	A	0.034	0.069	**PDE1A**	Human brain GWA traits include several cognitive traits and Alzheimers's. Mouse mutants have increased anxiety, abnormal open field behavior and hyperactivity^10^. There is extensive knowledge of PDE1A in brain biology and pathophysiology in humans and mice
chr36.25648690	7.98E−03	**4.05E−08**	C	0.034	0.069		

^1^RM or RW GWA-test, with multiple testing correction applied for all tests; genome wide significant in bold, suggestive underlined (see Results, Methods).

^2^Gene annotation of gene(s) nearest single SNPs or spanned for SNP intervals; SNPs within a gene in bold, high brain relevance in any species underlined.

^3^Three Mb interval is syntenic to three unliked loci in humans. MC2R is predominantly expressed in the adrenal gland.

^4^Sources of brain enrichment data: mouse BioGPS, human GTEx. Human ABHD3 introns have eQTLs for other genes, including ROCK1 in cortex and ESCO1 in thyroid. Mouse ROCK1 ref., Greathouse, K.M. Brain Structure and Function 223: 4227–4241 (2018).

^5^KCNJ2 mutation and Andersen-Tawil syndrome description, OMIM170390. Note abutting gene KCNJ16 is enriched in thyroid.

^6^Many genes at this locus are expressed in salivary glands.

^7^Near GNPTAB; in human, proximal brain eQTLs for GNPTAB and CHPT1.

^8^ENSCAFG00000004229 is highly and widely expressed, including in the brain, appears to be a GLUD1/2 retroposed gene.

^9^The brain-enriched gene RP11-731C17.2 (ENSG00000273486.1) is near. Mouse ref. Diab, A. Neuroscience, 448:107–125 (2020).

^10^Data source: International Mouse Phenotyping Consortium.

By the RM test, two intervals were strongly associated with behavioral elimination, chr13:55,534,649–59,902,870 (4.37 MB) and chr1:22,989,459–25,289,424 (2.30 MB); and single markers at chr7:66,358,701 and chr19:21,040,815 were also significant (CanFam3.1 coordinates). By the RW test, the same regions on chromosomes 13 and 1 were strongly associated, in addition to single markers chr6:76,632,282, chr36:25,252,101, and chr15:40,757,218. As expected, there was little overlap between the loci detected by RM and RW. The two tests had similar yields and numbers of loci with single vs multiple SNPs. None of the significant or suggestive loci mapped for behavioral elimination here overlapped C-BARQ behavioral GWAS loci or evolutionary selection regions reported for UK Labrador Retrievers^12,27^. Comparison to behavioral and cognitive markers previously mapped across dog breeds also showed no overlap with the present findings.

We created comprehensive models to simultaneously determine the effect sizes of candidate loci (Table 4). For RM loci, the effect of the chr7:66,358,701 AA allele was so large that it overwhelmed all others. For RW loci, the chr3:47,134,935 AA allele also had a great effect, but its estimation is uncertain due to quasi-complete separation (due to low A allele frequency). Quasi-complete separation occurs when levels in a dependent variable separate an independent variable perfectly. When this happens, the estimate can be assumed to be very large, but its numeric estimator is unreliable. The RW loci chr13:57,789,399 and chr36:25,252,101 had large effects for the heterozygous state (odds ratio, OR = 14.42 and 5.05, respectively), but the homozygous risk state was absent in the cohort. In a combined RM and RW assessment, only chr3:47,134,935 and chr36:25,252,101 remained significant with large effect sizes (quasi-complete separation for chr3:47,134,935 and OR = 4.63 for chr36: 25,252,101). The effects detected for chr7 in RM in and for chr13 in RW became non-significant.

### Candidate gene annotation for theory building

Several loci implicate one or few genes positionally (Table 2). Some single-SNP loci lie within or near one gene, and one multi-SNP locus overlaps a single gene. Such candidate genes known to have major behavioral effects in other species include *CHD2*, *NRG3* and *PDE1A*. Exclusion of puppies for behavioral reasons prior to entering training is a complex trait likely to involve many brain functions. That and the small number of candidate genes suggests geneset enrichment analysis is unlikely to be useful here. While brain expression of candidate genes is consistent with this behavioral trait, at least 80% of mammalian genes are expressed in the brain. We thus avoid using the known biology of candidate genes to support the mapping and allow interpretation of our GWA candidate genes. However, for purposes of prioritization and theory building, we performed a survey of brain relevance and genomic demographics (Table 3). Four genes each were associated with human educational attainment and intellectual disability, of which *CHD2*, *NRG3* and *LRRC7* were associated with both. *NRG3* and *LRRC7* were also associated with accelerated divergence in humans. Very few candidate genes were implicated in human neurodevelopmental disorders (N = 2 for high confidence geneset), autism (N = 1 for known and suspected) or epilepsy (N = 2). One gene, *CHD2*, was associated with all intelligence and neurodevelopmentally related traits mentioned above. *CHD2* was also the only tier 1 candidate known to be a haploinsufficient disease gene and to be intolerant to loss of function mutation. *DRAM1* is notable because it is among the genes most strongly associated with structure of many brain regions across seven studies (GWAS Catalog). For example, *DRAM1* was mapped with a P = 5 × 10^−52^ in a GWAS of sub-cortical volume^31^.

**Table 3:**
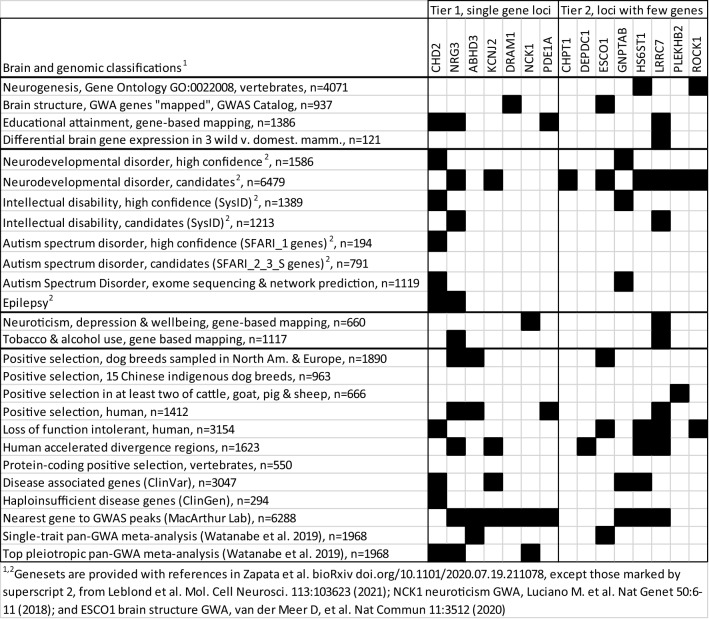
Brain trait and genomics demographics of behavioral elimination GWA candidate genes.

For two loci that have more than one candidate gene, this analysis together with the behavioral relevance noted in Table 2 implicate one gene at each locus: *LRRC7* on chr6 and *HS6ST1* on chr19. *HS6ST1* was one of only two candidates known to be a curated neurogenesis gene and is related to *HS6ST2*, which we previously mapped for increased social behavior. Although *ABHD3* was classified as a positional single-gene locus, the gene harbors expression quantitative trait loci (eQTLs) for *ROCK1* and *ESCO1*; and all three genes are good behavioral candidates (Tables 1 and 2). Two loci contain 66 other candidate genes that were not analyzed, but which contain genes with established (e.g., the neurodevelopmental genes *SMAD4* and *EPHA5*; and *MC2R*, the adrenocorticotropic hormone receptor expressed in the adrenal gland which receives the last signal within the hypothalamic, pituitary, adrenal axis) or more enigmatic (e.g., *CCN2*/*CTGF*^32^) behavioral relevance.

## Discussion

### Experimental approach and robustness

Our genome scan of risk of elimination due to undesirable behavior in Labrador Retrievers in the TSA detection dog breeding and raising program yielded six genome-wide significant and five suggestive loci. As far as we know, none of the mapped haplotypes has been reported in previous genetic mapping studies or genome scans for footprints of selection under domestication. Our mapping approach was to use the ROADTRIPS method developed for case–control association testing in related individuals sampled from structured populations. The method is not constrained by how subjects are related and allows for inclusion of individuals with pedigree and phenotype data but lacking genotypes. The PCA analysis distinguished the slight difference that resulted from the geographical origins of breeding Labrador Retrievers from the US and Australia. However, the two Labrador Retriever populations are closely related and US dogs contributed to the Australian program^28^. That was supported by the STRUCTURE analysis, which failed to detect population structure in our cohort. The Q-Q plots showed good control of the false positive rate due to population structure and family relatedness (consistent with studies of ROADTRIPS’ control of such type 1 error in case–control family-based studies^30^). A limitation of the cohort and the indicated mapping approach is the inability to calculate heritability. We were also not able to perform a type of validation using the same cohort because it could not be split into learning and testing sets (as the pedigree was included in the association analysis).

There are several challenges to the replication of this study related to the definitions of behavioral traits. Studies which overlapped the time of this work assessed the screening methods of the TSA^24,25^ and Australian Border Force Detector dog^26^ breeding and training programs. Based on survey data from 34 TSA dog handlers, 13 of 15 traits measured in TSA puppy testing showed content validity (i.e., the TSA standardized tests given to dogs in their first year matched well with handlers’ understanding of the most important operational traits)^25^. Unmeasured traits that were also predictive of success included “play” and off-duty “calmness”. However, for our cohort, the pretraining elimination of dogs for behavioral reasons was subjective and we do not have data describing the bases for those final decisions. Any behavior that is perceived as incompatible with successful training and deployment can result in elimination in olfactory detection programs^28^. Examples include, different types of fearfulness and anxiety, aggression, hyper- and hypo-activity, lack of innate drive to search and possess training toys, and insufficient human socialization. We don’t know how this information was used, but for some dogs eliminated due to behavior, our data noted traits such as distractibility, lack of motivation, and submissive urination. A recent study of 17 experienced explosive detection canine practitioners in the law enforcement, military, federal, and private sectors highlighted the following traits as being associated with success: “hunt drive” (motivation, a high level of energy, focus, and the ability to ignore or recover quickly from distractions), stamina to continue working, and the ability to generalize odors (e.g. identify explosives that are similar but not identical to those used in training)^33^. At the same time, behavioral tests, descriptive vocabulary, and other aspects of explosives dog selection are inconsistent between working dog organizations^34^. Such variable criteria for selecting dogs, as well as differences in work specialization and environments, may make it challenging to find genetic associations unless they have large effects and are correlated across multiple traits. Strengths of the present model include standardized TSA puppy program evaluation, pedigree information, and presence of vastly reduced genetic, and thus also phenotypic, heterogeneity within single breeds. In ongoing studies, we are analyzing the longitudinal TSA puppy testing data for these same dogs—separately and in combined analyses with the program elimination data reported on here.

### Implications for the working dog and behavioral genetics fields

Because Labrador Retrievers are very popular working dogs, an important finding is that behavioral haplotypes previously mapped or shown to be under selection in this breed^12,27^ were not associated with pretraining elimination due to behavior. That was also true for large effect behavioral variation mapped in interbreed GWASs^13,14,35,36^. For instance, a haplotype with a coding variant of *IGSF1* that is strongly associated with anxiety and fear traits across breeds and has an allele frequency of 0.18 in pet Labradors^14^ was homozygous non-risk in the present cohort. These findings are consistent with effective negative selection of such variations in hunting line Labradors or in the breeding programs involved here. Our identification of new loci associated with pretraining elimination due to behavioral reasons suggests that selective breeding might be used to improve the success rates in working dog breeding populations. The replication of this study in other working dog populations will be critical to decisions about whether and how to incorporate these markers into breeding strategies. If genome wide polygenic risk could be measured in well-powered cohorts, genomic estimated breeding values built on that would likely surpass the efficiency of methods based on pedigree or phenotype^4^.

Joint modeling of mapped loci showed at least two that have moderate-to-large effect sizes. The chr36:25 Mb locus had an OR of 4.63 and chr3:47 Mb can be considered incalculable due to quasi-complete separation. Importantly, the chr3:47 Mb estimate was from comparing homozygotes states whereas there were no dogs homozygous for the elimination risk allele at the chr36:25 Mb locus. Further studies of all mapped haplotypes are necessary to understand the evolutionary, genetic, and physiological mechanisms, and relevance to other breeds. The rapid genetic divergence and population bottlenecks in the development of dog breeds over the last few hundred years have resulted in phenotypes with simple inheritance patterns that have been associated with different genetic variants between breeds^37^. However, complex behavioral traits are influenced by many genetic and environmental factors and thus are challenging to understand. Historically in animal breeding, quantitative traits were described by the “infinitesimal model” in which phenotypes result from large numbers of genetic factors, each with an infinitesimal additive effect. More recently, the “omnigenic model” describes two types of genetic effects^38^. Core variants have larger effect sizes and occur in biochemical pathways related to the phenotype, while peripheral variants have smaller effects. If additional investigation showed our candidate loci are characteristic of the omnigenic model, that would suggest the large effect variations may have diagnostic and interventional utility.

The present working dog model is part of an untapped birth to death cohort with standardized health, behavior, cognitive, training, performance, and environmental data. If the necessary resources were in place, molecular epidemiological and computational psychiatric approaches would present a powerful framework for studying learning and working performance as described in the Introduction. Although ethology requires deconstruction to describe and measure isolated effects, it is clear the complex behaviors studied here are integrated across physical and behavioral breed conformations, cognition, temperament, critical period development, and gene-environment effects. As an example of the problem, consider a study of over 11,000 cadets assessed throughout their time in the US Military Academy^39^. At entry, cognitive ability was negatively correlated with both physical ability and grit (i.e., zeal, hard work, and perseverance). However, whereas cognitive ability was the best predictor of academic and military grades, both completion of initiation training and 4-year graduation were predicted better by non-cognitive traits. We are not equating TSA dog training with West Point cadet training, but rather drawing attention to the limitation of studying subjects sampled at elite institutions. Compared to the total population, elite cohorts tend to have very low variances across desired traits (i.e., everyone is preselected from the tail of a distribution). Our goal here was to genetically identify the likelihood of success in entering TSA training. If we had instead mapped failure to graduate training (i.e., using only dogs who succeeded in entering training), it is unlikely we would have identified the loci we did. Moreover, a complete working dog model would allow life course studies across a 12-year mean lifespan.

### Gene annotation for gene prioritization, theory building and future validation

Because the mapped trait is complex and the GWA cannot be confirmed yet, the gene annotation provides little weight as evidence that the mapping is true. Without knowing that and whether variation affecting a candidate gene contributes to the phenotype, any biological interpretation of that gene is tentative. However, both the effect sizes of loci and the biology of candidates can be used to prioritize follow-up studies. Some investigators may want to pursue the largest effect loci for breeding purposes. Others may prioritize validating and dissecting the molecular mechanism of a candidate gene at a single-gene locus based on, say, known protein function, brain expression patterns, or human or mouse phenotypes (see brain relevance of candidate genes in Table 2). Candidate genes of interest include *CHD2*, *NRG3*, *DRAM1* and *PDE1A* among the tier 1 loci that positionally implicate one gene. The *CHD2* and *PDE1A* loci are also of interest for having large effect sizes in the combined RM/RW comprehensive model. The tier 1 locus implicating *ABHD3* is supported by the comprehensive RM model. Notably, human *ABHD3* contains variations associated with expression levels of nearby *ROCK1* and *ESCO1*, which are strong behavioral candidate genes. At two tier-2 loci implicating more than one gene, the biological relevance favors one gene at each locus: *LRRC7* on chr6 and *HS6ST1* on chr19. *LRRC7* is particularly interesting here because it is associated with human educational attainment; intellectual disability; neuroticism, depression, and subjective wellbeing; and tobacco and alcohol use. It has also been shown to be under positive selection in humans and to be differentially expressed in three pairs of mammals comparing wild vs. domesticated species^40^. *HS6ST1* is interesting because we previously mapped canine social behavior to the locus of its paralog *HS6ST2*, which is also a neuroticism GWA gene in humans^14,41^.

The extensive mapped intervals on chr1 and chr13 are suggestive of recent positive selection. Of those, the hits on chr13 are also supported by the comprehensive RW model (Table 4). Both loci contain at least one prominent neurodevelopmental gene (*SMAD4* and *EPHA5*, respectively) and one neuropeptide receptor central to the Hypothalamic–Pituitary–Adrenal/-Gonadal axes (melanocortin/adrenocorticotropic hormone receptor *MC2R*, adrenal/HPA; and gonadotropin-releasing hormone receptor *GNRHR*, pituitary/HPG). A coding variant of *MC2R* common in ancient and herding dog breeds (~ 25% and ~ 8% allele frequencies, respectively), but absent or rare in other breeds including Labrador Retrievers, was shown to be associated with reduced gazing at experimenters in the “unsolvable test”^42^. Of our three mapped SNPs in the interval containing *MC2R*, the nearest to that variant position is 538,389 bp away (the others ~ 1 Mb or more). That coding variant and our risk allele at the nearest SNP are both present in the canFam4 German Shepherd genome assembly (alleles A and G, respectively; compared to alleles G and A in the Boxer assembly canFam3.1). Further studies are necessary to determine if the haplotype we mapped contains this *MC2R* coding variant.

**Table 4:** Comprehensive modeling for simultaneous effect size determination of GWAS hits.

Chromosome	Position (CanFam3.1)	Allele comparison	RM			RW			RM and RW		
			Odds ratio	95% Wald confidence limits		Odds ratio	95% Wald confidence limits		Odds ratio	95% Wald confidence limits	
1	2,52,89,424	AA vs. GA	0.67	0.06	7.491	–	–	–	0.173	0.001	20.159
3	4,71,34,935	AA vs. GG	–	–	–	> 999.999	39.792	> 999.999	> 999.999	16.219	> 999.999
3	4,71,34,935	AG vs. GG	–	–	–	0.496	0.124	1.986	0.465	0.111	1.939
4	3,14,20,247	AG vs. GG	1.282	0.293	5.613	–	–	–	1.052	0.143	7.725
6	7,66,32,282	AG vs. GG	–	–	–	0.365	0.034	3.956	0.031	< 0.001	8.034
7	6,63,58,701	AA vs. GG	112.194	8.222	> 999.999	–	–	–	247.529	0.408	> 999.999
7	6,63,58,701	AG vs. GG	0.741	0.195	2.818	–	–	–	1.724	0.35	8.484
9	1,65,59,175	AA vs. GG	–	–	–	1.804	0.153	21.317	1.179	0.073	18.961
9	1,65,59,175	AG vs. GG	–	–	–	0.069	0.008	0.574	0.081	0.009	0.699
13	5,77,89,399	AC vs. CC	2.014	0.238	17.033	14.416	1.482	140.223	8.144	0.306	216.926
15	4,07,57,218	AA vs. GA	–	–	–	2.016	0.265	15.34	7.371	0.235	230.76
19	2,10,40,815	AA vs. GA	1.087	0.288	4.098	–	–	–	1.195	0.217	6.598
23	3,33,69,350	AA vs. GG	–	–	–	2.737	0.246	30.482	4.925	0.188	129.167
23	3,33,69,350	GA vs. GG	–	–	–	0.206	0.008	5.551	0.295	0.007	13.169
36	2,52,52,101	AC vs. CC	–	–	–	5.055	1.541	16.581	4.631	1.376	15.588

Positions are generated from the CanFam3.1 genome assembly.

It will be interesting to see if closely related breeds, such as Golden and Flat Coated Retrievers, or more distantly related working dog breeds like German Shorthaired Pointers carry the risk alleles we mapped on chr1 and chr13 and can thus be used for fine mapping^43^. Alternatively, brain eQTL data for Labrador Retrievers could reveal if any genes in the intervals have differential expression associated with that haplotype^44^. Whereas that requires postmortem samples, it is also possible to validate mapped loci behaviorally, especially where both risk and non-risk alleles are common. For some candidate genes, there are testable clues of associations with one type of brain trait, such as *NCK1* with neuroticism, and *DRAM1* and *ESCO1* with brain structure. For instance, the *NCK1* locus can be explored for association with related traits in genotyped dogs with C-BARQ dog owner behavioral questionnaire data^36^. *DRAM1* and *ESCO1* can be tested for genetic associations with MRI-based brain structure differences^16,35^. Lastly, the several dog loci that in humans are associated with cognitive traits can be tested for epistasis with each other and for association with the TSA training test data for this population that is currently being analyzed (and with emerging canine cognitive tests^11^).

## Conclusions

Our genome scan of pretraining elimination due to undesired behavioral traits in the TSA detector dog breeding and training program identified six genome wide significant loci. We used family-based association methods that controlled for relatedness by inclusion of the pedigree as a covariate and increased power by inclusion of dogs with phenotype but no genotype data. The top limitation of the study is the current lack of a validation cohort, which we are addressing in ongoing studies. One suggestion that the mapping is likely to be true is the strong behavioral relevance of multiple candidate loci which implicate a single gene. These findings are consistent with the possibility of improving the efficiency and quality of working dog programs through genomic estimated breeding values.

## Materials and methods

### Data acquisition

Data used in this study were generated from an olfactory detection dog breeding and training program the TSA ran from 2002 to 2013. Samples from litters born between 2002–2012 were genotyped in 2012–2013. The behavioral data were stored in a RedCap^45^ database that was designed specifically for genomic and behavioral research. Phenotype data for 528 Labrador Retrievers were provided. In that original study, 296 of the same dogs were randomly selected for genotyping (Illumina Infinium Assay and CanineHD Beadchip (Illumina Part No. 11322460)) and the resulting information was included in the data we received. That genotyping data yielded 173,662 SNPs spanning the dog genome.

### Ethics statement

The biological samples and genotype data, and the phenotype data were collected within the US Transportation Security Administration’s (TSA) canine olfactory detection breeding and training program between 2002–2013 following all necessary guidelines and regulations. The present study is based on access to those data.

### Data processing and statistical analysis

Genotyping data were first converted from the CanFam2.1 to CanFam3.1 dog genome build using the UCSC Lift Genome Annotations tool (http://www.genome.ucsc.edu/cgi-bin/hgLiftOver). Quality control was performed in PLINK 1.90^46^ using a minor allele frequency cutoff of 2.5%, Hardy–Weinberg equilibrium filter of 1E-5, and maximum SNP missingness rate of 10%. This resulted in a cleaned dataset of 112,284 SNPs.

To investigate underlying population structure, a Principal Components Analysis (PCA) was performed on the genotyped dogs after imputing missing genotypes and clumping SNPs utilizing the *bigsnpr* package in R^47^. Variance explained for each included SNP was exported for the top 25 PC’s to assess their loading values. A pedigree for the family of dogs was developed using the *kinship2* package in R^48^.

To identify genomic regions associated with behavioral elimination, a Genome Wide Association Study (GWAS) was performed using *ROADTRIPS2*^30^. Prior to running, kinship coefficients^49^ were calculated using the *KinInbcoef* v1.1 software^50^ and missing genotypes generated for non-genotyped, related dogs with known elimination status. *ROADTRIPS2* was run with both genotyped and non-genotyped dogs that shared the same pedigree and had elimination status. Figures were generated for the output utilizing the *qqman* package in R^51^. Genome wide significance was based on Bonferroni adjustment (α = 0.05/112,246 SNPs/2 GWAS tests = 2.2 × 10^−7^) and suggestive significance was arbitrarily set at P ≤ 1 × 10^−5^.

The comprehensive models for simultaneous effect size determination were performed on genotyped dogs alone using only the significant hits generated by the GWAS. This approach was carried on as multiple logistic regressions where the elimination status was defined as the dependent variable and the specific alleles for the selected hits defined as categorical dependent variables. This approach was done independently for hits obtained through RM and RW statistics in addition the combined RM and RW list. All regression analysis was performed in SAS/STAT v.9.4.

### Genome annotation

Genome annotation was performed on the UCSC Genome Browser. All canine genome coordinates reported here correspond to the canFam3 assembly. Gene annotation was performed using the Broad Improved Canine Annotation v1^52^ and checked for different or missing gene content and accepted gene nomenclature^53^ by analyzing the syntenic intervals in the human genome (In Other Genomes (Convert) function; hg19 and hg38 assemblies).

## Supplementary Information

Supplementary Figure S1.

## Data Availability

Phenotype data are included as Supplementary Information in this work. Genotyping data are available in a public repository: https://data.mendeley.com/, 10.17632/hrtpmfyypm.1.
