# Supplementary material for: Genome scanning of behavioral selection in a canine olfactory detection breeding cohort
Source: Sci Rep. 2022 Sep 2;12:14984. doi: 10.1038/s41598-022-18698-4 (PMC9440224; doi:10.1038/s41598-022-18698-4)
Supplement: Supplementary file 1 — Supplementary Figure S1. [file 41598_2022_18698_MOESM1_ESM.pdf]

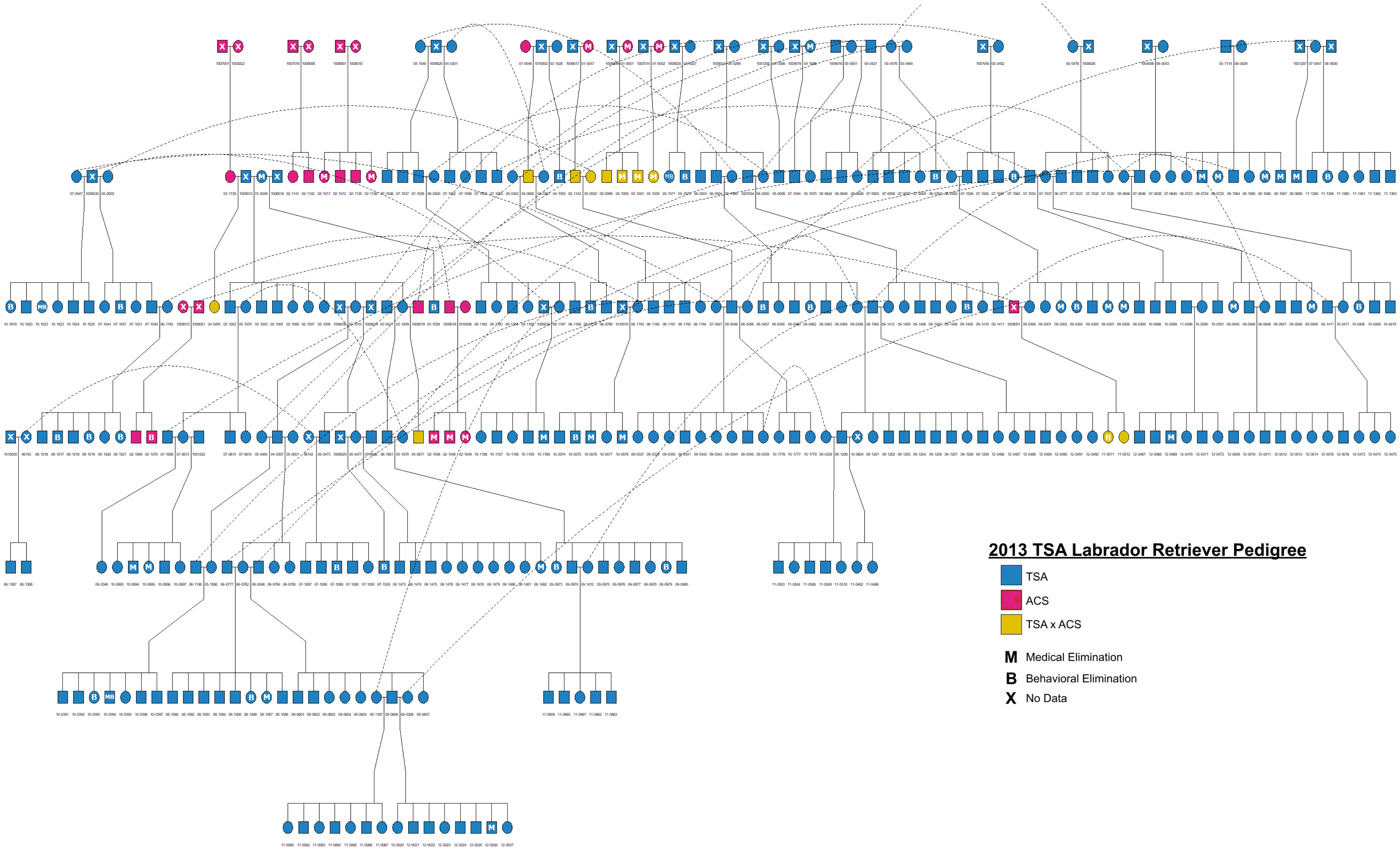

Figure S1. Pedigree of this study's TSA breeding program cohort. ACS dogs refer to dogs sourced from the Australian Customs Service. Dogs are classified for program elimination for medical or behavioral reasons.
